# Atlantic Salmon (*Salmo salar*) GILL Primary Cell Culture Oxidative Stress and Cellular Damage Response Challenged with Oxytetracycline Antibiotic

**DOI:** 10.3390/toxics13110914

**Published:** 2025-10-24

**Authors:** Luis Vargas-Chacoff, José Ramírez-Mora, Daniela Nualart, Francisco Dann, José Luis P. Muñoz

**Affiliations:** 1 Laboratorio de Fisiología de Peces, Instituto de Ciencias Marinas y Limnológicas, Universidad Austral de Chile, Valdivia 5090000, Chile; jnobelrm@gmail.com (J.R.-M.); daniela.nualart@gmail.com (D.N.); franciscojavierdann@gmail.com (F.D.); 2 Millennium Institute Biodiversity of Antarctic and Subantarctic Ecosystems, BASE, University Austral of Chile, Valdivia 5090000, Chile; 3 Centro FONDAP de Investigación en Dinámica de Ecosistemas Marinos de Altas Latitudes (IDEAL), Universidad Austral de Chile, Valdivia 5090000, Chile; 4 Integrative Biology Group, Valdivia 5090000, Chile; 5 Programa de Doctorado en Ciencias de la Acuicultura, Escuela de Graduados, Universidad Austral de Chile, Puerto Montt 5480000, Chile; 6 Centro de Investigación y Desarrollo I~Mar, Universidad de los Lagos, Puerto Montt 5480000, Chile

**Keywords:** antibiotics, Atlantic salmon, gills, oxidative stress, ROS

## Abstract

Salmon farming has been affected by various bacterial diseases, and the use of antibiotics (such as oxytetracycline “OTC”) to control these diseases has become necessary and thus routine. This study aimed to determine how the gill cells are affected by OTC in *Salmo salar*. Gill tissue culture was performed in periods of 0.5, 1, 3, 6, 12, and 24 h, assessing the enzymatic activity and mRNA expression of catalase (CAT), cytochrome p450, glutathione peroxidase (GPx), glutathione reductase (Gr), and superoxide dismutase (SOD), HSP70 and HSP90, in response to two doses of OTC: 0.25 (low), and 3 µL/mL (high). The results indicated that the enzymatic activity of SOD and CAT showed low enzyme activity at both doses. At the same time, GR presented varied response patterns depending on the time and dose of OTC used, contrary to GPx, which just increased the enzyme activity at early times. Although the mRNA expression presented the most precise pattern of expression, they were not in line with the enzymatic activities. The HSP70 and HSP90 mRNA expression response (as a cellular damage marker) increased mRNA levels at low and high doses, respectively, but at different times, alluding to a differentiated response given by the size of the chaperone. These results suggest an oxidative response of the gills to OTC exposure and constitute significant information on the amount of OTC used in aquaculture and on methods for improving the optimal dose of drugs, fish health, and, consequently, environmental health.

## 1. Introduction

The gills are a multifunctional tissue, serving relevant functions such as breathing, osmo-regulation, and ionic balance, and also play an important role in several processes during the ontogeny stage, including smoltification [1]. In addition, the gills have direct contact with water. With this connection, they suffer from many influences, such as temperature; a master abiotic factor; as well as salinity; pH; and pollutants [1,2,3,4,5,6,7,8,9].

**Figure 1:**
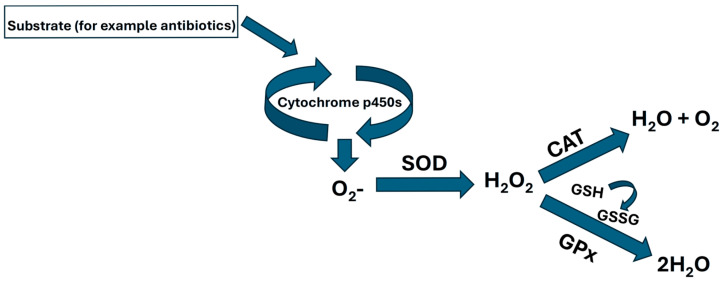
Schematic pathway of oxidative stress.

According to several functions mentioned earlier, there are diverse responses; one of greater relevance is the oxidative stress response [10], which is due to the animal’s need to remove toxins from various tissues, such as the gills (Figure 1).

Chronic toxicity assays evaluate the harmful effects of toxicants on organisms after extended exposure to sublethal concentrations [11]. The responses that can be evaluated include physiological, histological, and molecular alterations, including others [12]. Some of the sublethal responses that can be assessed are described below:

I. Detoxification enzymes. These are enzymes responsible for transforming toxic compounds into compounds that are easily eliminated by organisms. Alterations in the levels or activity of these enzymes are one of the most sensitive sublethal responses and indicate an alteration in the organism due to exposure to a toxicant. Enzymes can be categorized based on the metabolic phase in which they operate as described below:

- Phase I or oxidative-phase enzymes. Cytochrome p450 is the largest enzyme complex involved in this phase of metabolism. In general terms, we can say that Phase I is a set of oxidation reactions that prepare toxicants for transformation by Phase II reactions.
- Phase II enzymes. The result of these reactions is a significant increase in the water solubility of the xenobiotic, facilitating its excretion. One of the enzymes involved in this phase is glutathione S-transferase (GST) [13,14].

II. Oxidative stress parameters. Reactive oxygen species (ROS) are oxygen ions, free radicals, and inorganic and organic peroxides. They are generally tiny molecules that are highly reactive due to the presence of an unpaired valence electron shell. These species form naturally as a byproduct of normal oxygen metabolism; however, under certain circumstances, their levels can increase and cause damage to cellular structures. This leads to a situation known as oxidative stress, characterized by enzyme activation/inhibition, lipid peroxidation (LPO), DNA (deoxyribonucleic acid) damage, and ultimately, cell death. The most affected enzyme activities, which are, therefore, used as biomarkers, are glutathione reductase (GR), glutathione peroxidase (GPX), and catalase (CAT), among others [14,15,16].

The response to toxic compounds has been tested in various animal and fish models, especially those of commercial importance. However, there are toxic effects of compounds used in aquaculture, namely antibiotics. These are used to control and combat bacterial diseases, which cause many problems in fish farming [17]. In Chile, the farming of Atlantic salmon stands as a crucial economic activity for the nation. However, numerous bacteria are present, resulting in significant financial losses. Therefore, the use of antibiotics has been constant, with florfenicol and oxytetracycline (OTC) being the most commonly used [18]. OTC is broad-spectrum with bacteriostatic action. OTC is a compound that inhibits protein synthesis in the 30S subunit of the bacterial ribosome, preventing bacterial replication and then inducing their death [19,20,21]. However, in vitro and ex vivo studies have indicated physiological, neuroendocrine, and immune changes, causing an increase in the oxidative response or a decrease in the immune response [9,22,23]. The objective of this study was to determine the oxidative response and cellular damage in Atlantic salmon gills’ primary cell culture. To do this, primary cell cultures of gills were prepared and subjected to various concentrations of OTC in a time-kinetics study. Changes in gill superoxide dismutase (SOD), glutathione peroxidase (GPx), glutathione reductase (GR), catalase CAT, cytochrome p450, HSP70, and HSP90 were monitored. Our study helps us to better understand the use and abuse of antibiotics in aquaculture and how to plan monitoring using other tissues as targets.

## 2. Materials and Methods

### 2.1. The Ethics Statement

All experimental procedures adhered to regulations for the use of laboratory animals set forth by the Chilean National Commission for Scientific and Technological Research (ANID) and the Universidad de Los Lagos (Res. # 01/2023).

### 2.2. Sampling Procedure

For in vitro experiments, healthy specimens of Atlantic salmon (*Salmo salar*), weighing approximately 500.32 ± 12.4 g, were obtained from Unidad de Produccion Acuicola (Universidad de Los Lagos) fish farm (Rupanco Lake, Chile) and then were transported to the laboratories of the Faculty of Science (Universidad Austral de Chile, Valdivia, Chile). For the extraction of gill tissue, the fish were sedated using a lethal dose of 2-phenoxyethanol (1 mL/L), following the protocol of Vargas-Chacoff et al. [4].

### 2.3. Primary Culture

For primary culture, small pieces of gill tissue (approximately 10–20 mg) were obtained from *S. salar* gill explants and removed under aseptic conditions [24]. The primary gill cell cultures used in this study were validated following established methodologies to ensure growth, viability, and physiological stability throughout the experimental period. Cell viability and morphology were routinely monitored under an inverted microscope (AE31E Inverted Microscopes, Motic, Kowloon, Hong Kong) to confirm the maintenance, absence of contamination, and typical explant morphology [24]. The gills were utilized for primary cell culture, which was seeded, maintained in a six-well plate, and incubated at 18 °C in an air atmosphere for a minimum of 24 h [24]. The medium used for cell and tissue culture was Leibovitz’s 15 (L-15), with each well containing 1 mL of L-15. This medium was improved by adding 10% fetal bovine serum (FBS) from Invitrogen Gibco (Thermo Fisher, Waltham, MA, USA), following the protocols of Pontigo and Vargas-Chacoff [25] and Nualart et al. [26]. For kinetic experiments at 0.5, 1, 3, 6, 12, and 24 h at 18 °C, 1 μL/well of the antibiotic solution was added. Control plates had the same volume of medium without the antibiotic. All experiments were conducted in triplicate and repeated twice independently.

### 2.4. In Vitro Experimental Treatment

Twenty-four hours after seeding each portion of the gills, the medium was changed for testing at different doses of oxytetracycline (OTC) (Merck, Darmstadt, Germany; CAS No: 79–57-2): 0.25 μg/mL, 3 μg/mL, and a control group. The doses of OTC selected are akin to those outlined by Tafalla et al. [27].

### 2.5. Extraction of Total RNA from Gill Cells

After the kinetic experiments were concluded, the cell culture medium was discarded, and 500 μL of TRIzol reagent (Sigma, St. Louis, MO, USA) was introduced to collect the cells. These cells were then frozen using liquid nitrogen to prepare for RNA extraction. The total RNA was extracted using TRIzol reagent per the manufacturer’s instructions and stored at −80 °C. Concentration of RNA was determined at a wavelength of 260 nm using a NanoDrop spectrophotometer (NanoDrop Technologies, Wilmington, DE, USA). The RNA samples’ integrity was assessed using 1% agarose gel electrophoresis. For the reverse transcription process, 2 μg of RNA was used as the template to synthesize cDNA. This procedure followed standard protocols, using MMLV reverse transcriptase from Promega (Promega, Madison, WI, USA) and an oligo-dT primer from Invitrogen (Invitrogen, Waltham, MA, USA).

### 2.6. qRT-PCR Analysis

The experiments were conducted using the AriaMx real-time PCR System (Agilent, Santa Clara, CA, USA). cDNA was prepared at a concentration of 100 ng, which served as the template for the PCR. These reactions utilized Brilliant SYBR Green qPCR reagents (Stratagene, La Jolla, CA, USA). Each assay was performed in triplicate with a total reaction volume of 14 μL, consisting of 6 μL of SYBR Green, 2 μL of cDNA (100 ng), 1.08 μL of a primer mixture, and 4.92 μL of PCR-grade water.

The PCR process started with an initial denaturation at 95 °C for 10 min, followed by 40 cycles that included denaturation at 95 °C for 10 s, annealing at 60 °C for 15 s, and extension at 72 °C for 15 s. To verify the specificity of the amplified products, a melting-curve analysis was conducted after amplification.

The expression levels of genes such as cytochrome p450 (p450), glutathione reductase (GR), glutathione peroxidase (GPx), superoxide dismutase (SOD), heat shock protein 70 (HSP70), and heat shock protein 90 (HSP90) were measured using the comparative Ct method (2^−ΔΔCT^) [28]. The results are expressed as fold changes compared to the endogenous reference gene 18S and are relative to control cells that were not stimulated. The sequences of the primers used in this research are provided in Table 1, and their efficiencies were determined using the Rasmussen formula [29].

### 2.7. Antioxidant Enzyme Activity

#### 2.7.1. Homogenization

The gill was homogenized in a 100 mM phosphate buffer at pH 7.4, which contained 0.15 M KCl, 1 mM EDTA, 0.1 mM PMSF, and 1 mM DDT in a 1:4 ratio (sample weight to volume buffer). The samples were centrifuged at 14,000 rpm for 30 min, with the temperature held at 4 °C. The resulting supernatants were then utilized to assess the enzymatic activity. These measurements were conducted in triplicate using a spectrophotometric analysis with a microplate reader (MultiscanGo, Thermo Scientific, Waltham, MA, USA) and analyzed with ScanIT 3.2 MultiscanGo software. Enzymatic activities were measured at 20 °C and expressed in substrate moles converted to product per minute. Specific enzymatic activities are expressed according to the protein concentration in each sample (mU/mg or U/mg).

#### 2.7.2. Specific Enzymatic Conditions

Catalase (CAT) activity was assessed following the methodologies described by Aebi [33] and Lopez-Galindo et al. [34]. The decomposition of H_2_O_2_ was monitored at 240 nm for a duration of five minutes. One unit of catalase (CAT) activity is defined as the amount of H_2_O_2_ broken down per minute for each milligram of protein.

Glutathione reductase (GR) catalyzes the conversion of oxidized glutathione (GSSG) to its reduced form (GSH). This process involves the enzyme glutathione peroxidase (GPx), which reduces peroxides and lipid hydroperoxides. This activity is quantified in terms of mmol/min/mg of protein. It was performed at 340 nm over 5 min, monitoring the oxidation of NADPH following the procedures laid out by Carlberg and Mannervik [35], and Lopez-Galindo et al. [34].

Glutathione peroxidase (GPx) catalyzes the reduction of hydrogen peroxide and lipid hydroperoxides to safer compounds using reduced glutathione (GSH). This process is evaluated by measuring the oxidation of NADPH at an absorbance wavelength of 340 nm, as described in the studies by Flohe and Gunzler [36] and Lopez-Galindo et al. [34]. In parallel, superoxide dismutase (SOD) activity is evaluated based on its capacity to catalyze the dismutation of the superoxide anion (O_2_^−^) into hydrogen peroxide (H_2_O_2_) and molecular oxygen (O_2_). Following the protocol established by Sun et al. [37], SOD activity is assessed by measuring its inhibitory effect on the reduction in nitroblue tetrazolium (NBT) to formazan. This inhibition is quantified spectrophotometrically at 560 nm over a 30 min incubation period.

#### 2.7.3. Protein Quantification

The study utilized the bicinchoninic acid (BCA) method and employed the BCA Protein Assay Kit (Pierce #23225, Darmstadt, Germany). All assessments were carried out using a MultiscanGo Microplate Reader (Thermo Scientific) in conjunction with ScanIT 3.2 software.

#### 2.7.4. Statistical Analyses

Data are presented as averages along with the standard error of the mean (S.E.M.). The effectiveness of the polymerase chain reaction (PCR) was assessed through linear regression analysis using LinRegPCR (Microsoft Excel software 365, version 2508). A two-way ANOVA was performed to analyze gene expression, considering different stimuli and time as factors contributing to variance, and also the interaction between both factors. Before conducting the ANOVA, the data were checked for normality and homoscedasticity. Following the ANOVA, Tukey’s HSD test was conducted as a post hoc analysis. All statistical analyses and graphs were created using GraphPad Prism 9 software.

## 3. Results

**Figure 2:**
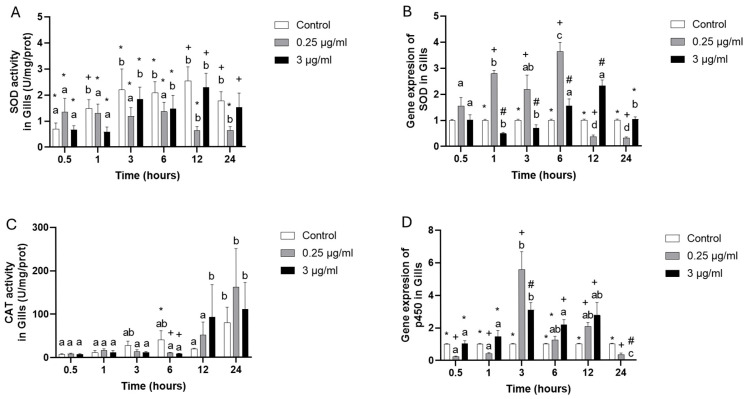
Gill cell culture treated with oxytetracycline “OTC”. Enzyme activity in (**A**) SOD, and (**C**) CAT; and gene expression in (**B**) SOD, and (**D**) p450. The relative expression of genes was calculated by the comparative Ct method (2^−ΔΔCT^) using the 18s ribosomal protein as the internal reference gene. Each value represents the mean ± S.E.M. Letters indicate statistical differences among the same treatment at different time points, and statistical differences over time points. Symbols (*, #, and +) indicate statistical differences among different treatments at the same time point (two-way ANOVA, *p* < 0.05).

The SOD (superoxide dismutase) enzyme activity at 0.25 µg/mL showed the lowest activity during the last time points, 12 and 24 h; meanwhile, the 3 µg/mL did not show changes in comparison to the control group, indicating that it is dose-dependent. The gene expression of SOD at 1, 3, and 6 h resulted in an up-regulation at 0.25 µg/mL, but at 12 and 24 h showed a down-regulation, while at 3 µg/mL, indicated that at 1 and 3 h a down- regulation had occurred, while at 6 and 12 h an up-regulation were presented in comparison to control group (Figure 2A,B).

Catalase enzyme activity (CAT) decreased at both OTC concentrations at 6 h, but at 12 and 24 h presented elevated activity with high variability but did not present statistical differences (Figure 2C). The p450 (cytochrome p450) gene expression initially showed down-regulation at 0.5 and 1 h at 0.25 µg/mL. However, at 3 and 12 h, the highest levels of expression were presented, and a new down-regulation was observed at 24 h, indicating an “inverted U form” of expression. Similarly, a pattern was presented at 3 µg/mL, where 3, 6, and 12 h presented the highest levels of expression, having equal (0.5 and 1 h) or down-regulated expression at 24 h compared to the control group (Figure 2D).

**Figure 3:**
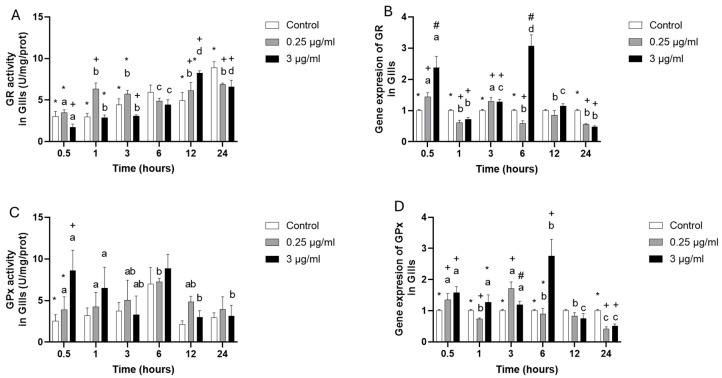
Gill cell culture treated with oxytetracycline “OTC”. Enzyme activity in (**A**) GR, and (**C**) GPx; and gene expression in (**B**) GR, and (**D**) GPx. The relative expression of genes was calculated by the comparative Ct method (2^−ΔΔCT^) using the 18s ribosomal protein as the internal reference gene. Each value represents the mean ± S.E.M. Letters indicate statistical differences among the same treatment at different time points, statistical differences over time points. Symbols (*, #, and +) indicate statistical differences among different treatments at the same time point (two-way ANOVA, *p* < 0.05).

The GR (glutathione reductase) enzyme activity only increased at 1 h at 0.25 µg/mL (Figure 3A); meanwhile, at 3 µg/mL presented lowest activity at 0.5, 3 and 24 h, and increased at 12 h (Figure 3A), notwithstanding that the gene expression presented up-regulation at 0.5 and 3 h, but at 1, 6 and 24 h showed down-regulation at 0.25 µg/mL (Figure 3B), while 3 µg/mL indicated that at 0.5, 3, and 6 h it was up-regulated, but at 1 and 24 h was down regulated (Figure 3B).

The GPx (glutathione peroxidase) enzyme activity only presented increasing at 0.5 in 0.25 µg/mL and 3 µg/mL (Figure 3C), being very similar in all experimental groups to that of the control group. The GPx gene expression presented in 0.25 µg/mL at 0.5 and 3 h was the highest value. However, at 1 and 24 h, it showed the lowest level of expression compared to the control group. The 3 µg/mL group presented an up-regulation at 0.5, 3, and 6 and a down-regulation only at 24 (Figure 3D).

**Figure 4:**
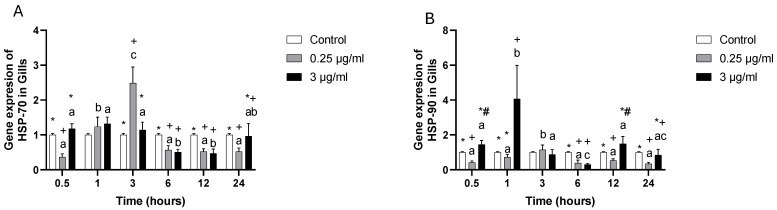
Gill cell culture treated with oxytetracycline “OTC”. Gene expression in (**A**) HSP70, and (**B**) HSP90. The relative expression of genes was calculated by the comparative Ct method (2^−ΔΔCT^) using the 18s ribosomal protein as the internal reference gene. Each value represents the mean ± S.E.M. Letters indicate statistical differences among the same treatment at different time points and statistical differences over time points. Symbols (*, #, and +) indicate statistical differences among different treatments at the same time point (two-way ANOVA, *p* < 0.05).

The HSP70 (heat shock protein) presented down-regulation at 0.5, 6, 12, and 24 h, having, only at 3 h, up-regulation in 0.25 µg/mL, while 1 µg/mL at 6 and 12 h presented down-regulation (Figure 4A). HSP90 gene expression presented down-regulation at 0.5, 6, 12 and 24 h in the 0.25 µg/mL group; in the meantime, the 3 µg/mL group showed up- regulation at 0.5, 1, and 12 h (Figure 4B).

## 4. Discussion

The disturbance or imbalance of the normal redox results in oxidative stress and is involved in many physiological problems and diseases. High ROS levels increase oxidative stress; it can, in turn, damage proteins and/or lead to downstream signaling in toxic pathways [38]. There are many studies about oxidative stress in mammals, especially in carcinogenics and aging [39] and also in fish with relation to aquaculture, for example, *Cyprinus carpio*, *Xiphophorus Helleri*, *Cirrhinus mrigala*, *Salmo salar*, *Dicentrarchus labrax*, *Oreochromis niloticus*, and *Oncorhynchus mykiss*, indicating that oxidative stress is a valuable biomarker to evaluate the toxic impacts of antibiotics or other pollutants on the health of fish [40,41,42,43,44,45,46].

According to the FAO 2024 report [17], aquaculture is expanding and becoming more intensive, leading to the increased use of antibiotics as disease-control mechanisms. A total of 463 tons of antibiotics were used in Chilean salmon farming, with an annual consumption of 0.47 Kg of antibiotics per ton of farmed salmon. Consequently, bacteria are more resistant [9]. In Chile, the OTC doses used in salmon farming diets are 75–100 mg/Kg, and in plasma levels of OTC, they are approximately 1 µg/mL. Our study demonstrated that the OTC antibiotic produced an effect on the gills’ oxidative stress response and cellular damage.

The O_2_^−^ can be derived from the detoxification phase of p450 where the cytochrome p450 complex cannot eliminate the toxic [38]. Our results showed an invert “U” form, indicating that at 3 h of exposure, the p450 is working at both doses (0.25 and 3 µg/mL). Curiously, in another study in liver primary cell culture of *S. salar* [9] with the highest doses of OTC, the response was later, being at 6 h in all doses (from 1 to 20 µg/mL), indicating a tissue- and dose-dependence. In addition, while in the liver of Swordtail fish (*Xiphophorus Helleri*), gene expression in the p450 isoforms (CYP1A, and CYP3A) showed up-regulation in male and female at 24–72 h of exposure to the antibiotic norfloxacin [43]. Due to their oxidation capacity, CYP enzymes play a significant role in phase I metabolism of drugs and xenobiotics, increasing the substrate’s polarity and aiding in excretion. Also, depending on the metal and the xenobiotic, different mechanisms were revealed as acting not necessarily at the transcriptional level, but primarily through post-transcriptional mechanisms which reduced the availability of heme groups or the functional activity of the protein without necessarily affecting the enhancement of mRNA expression induced by AhR agonists (AhR: aryl hydrocarbon receptor) [47,48,49,50,51]. Other antibiotics such as florfenicol produce metabolites that are harmful to the liver, causing injury, and leading to apoptosis and a loss-of function, resulting in irreversible damage at the cellular level and, therefore, at the individual level [52].

The superoxide radical (O_2_^−^) has toxic effects and needs antioxidants such as “SOD”, which is a metalloenzyme that is a part of the defense against O_2_^−^ [53]. SOD, in our study, presented two patterns: The enzyme activity showed a down-regulation of activities. In contrast, the gene expression showed an up-regulation early at low doses and late at high doses, compared to the control group. However, in the liver cell culture of *S. salar*, the response was overexpressed at 3 and 6 h in all experimental groups, indicating an antioxidant response [9]. In contrast, in mussels *Mytilus galloprovincialis* challenged with citalopram and bezafibrate with polyethylene microplastics, the SOD did not present variations [54]. Similar results were indicated in *Cyprinus carpio* following exposure to Benzethonium Chloride [40], contrary to *Cirrhinus mrigala*, where exposure to 1 μg/L and 1.5 μg/L of ciprofloxacin (it is a second-generation fluoroquinolone, a highly prescribed medication against various bacterial infections in humans and aquaculture practices [42]) presented high levels of SOD, being in line with high doses. Rodrigues et al. [55] determined that in rainbow trout, the SOD activity decreased, as in our results with OTC. This would indicate an inactivation of the antioxidant system produced by oxytetracycline. SOD would prevent lipid peroxidation, which could cause permanent damage to branchial cells. Lipid peroxidation is considered the initial step in cell membrane damage, for example, free radicals could provoke oxidative lipid degradation [56] and, as a consequence, cause muscle degradation, hemolysis, and nervous system and metabolic deterioration, causing multisystem damage that can lead to cell death [57,58].

As part of the signaling pathway, the catalase “CAT” takes the peroxide that comes from SOD and produces water and oxygen (Figure 1). López-Galindo et al. [34] and Álvarez-Muñoz et al. [59] in *Solea senegalensis* indicated a late elevation in branchial CAT activity when challenged with detergent, while in the tilapia nicolitica (*Oreochromis nilotica*) exposed to the anionic detergent SDS (sodium dodecyl sulphate), reported an inhibitory in vitro action on hepatic CAT [60]. Our results suggest that after 3 and 6 h of exposure, the CAT activity suffers an inhibition and, at 12 and 24, an elevation of activity, indicating that, potentially, what can be happening are the three following things: (A) a differential contribution is made by other antioxidants to remove H_2_O_2_; (B) the formation of ROS is occurring at different rates in each tissue; (C) the product (or any metabolites formed) may have a direct toxic effect on the gills. The enzymes GR and GPx are essential; GR acts as a scavenger for oxygen radicals, helping to reduce the oxidation of reduced glutathione (GSH) by facilitating the conversion of oxidized glutathione (GSSG) back into its reduced form, GSH. This conversion ensures adequate levels of GSH, which are crucial for glutathione peroxidase (GPx) [61]. In our study, the branchial cells presented similar patterns in both of the enzymes acting early. Equally, both exhibited a down-regulation of gene expression at the late kinetic stage. In the liver cells of Atlantic salmon treated with OTC, the GR activity and gene expression were lower than in the control group. In contrast, the GPx did not present a clear pattern of expression and enzyme activity [9].

However, the GR did not present any changes in the mussels *Mytilus galloprovincialis* challenged with citalopram and bezafibrate with polyethylene microplastics [54]. Gills of *Cyprinus carpio* exposed to Benzethonium Chloride did not present changes in either markers [40].

Our results between enzymatic activity and the expression of its underlying genes was not correlated or in line as expected. That the transcription (mRNA) goes to the translation into proteins, which, being non-coincident, was recorded by Vargas-Chacoff et al. [62] in *Sparus aurata*, where the study of gene and protein expression did not coincide, due to the lack of correlation between mRNA and protein expression, which is an indication of a failure of regulation at the level of mRNA synthesis, and protein translation, which could potentially be caused by post-transcriptional modifications not evaluated in this work affecting the protein synthesis.

The HSPs can be induced by temperature, but many studies have demonstrated that other factors can act as stressors and induce the HSPs [63]. These proteins can participate in the remodeling of damaged or misfolded proteins to conserve their native state, and can metabolize those that cannot be repaired, thus helping to maintain cellular function following stressful events [5,64,65,66,67,68,69]. In our study the chaperones HSP70 and HSP90’s expression presented two patterns, depending on the size of the HSP; the over-expressed response to 0.25 µg/mL at 3 h for HSP70; meanwhile, at 3 µg/mL, they presented an over-expression at 1 h in HSP90, suggesting that the early response is due to the high dose and large size of the chaperone HSP90, while the low doses need to be active for at least 3 h for chaperones with small sizes such as HSP70. Martínez et al. [69], in *Eleginops maclovinus*, challenged it with a high temperature and a bacterial injection, and they observed their overexpression in both of the chaperones at 4 and 8 days, respectively, while in *S. salar*, Vargas-Chacoff et al. [5] described high protein levels of HSP70 at 48 h in fish maintained in several salinities and thermal regimens.

## 5. Conclusions

The use of antibiotics in aquaculture is increasingly questioned, regulated, and is even being reduced due to problems with bacterial resistance and side effects, such as those described in our study.

This research helps us to understand that the excessive effect of antibiotics generates a physiological problem in fish, and that responses such as oxidative stress are activated to eliminate the products/metabolites generated by these disease treatments. These responses are dose-dependent, and time is also a factor to consider, as the signaling pathway must be activated. Also, our study demonstrated that gills can be a good target for studies of antibiotics’ effects, for considering the welfare of animals and to help us understand that use of antibiotics must be reduced.

## Figures and Tables

**Table 1:** Primer sequences for expression analysis. p450 (cytochrome p450), GR (glutathione reductase), SOD (superoxide dismutase), GPx (glutathione peroxidase), HSP70 (heat shock protein 70), HSP90 (heat shock protein 90), and 18S (18 subunit ribosomal).

Primer	Nucleotide Sequences (5′→3′)	Efficiency (%)	GenBank No/Reference
p450Fw	TCGTTCCTTGTCCGAAAGCAGA	100.4	Pedro et al., 2019 [30]
p450 Rv	TGTCGGTACCAGCACCAAACAT		
GR Fw	AAAGTGCCAGTACCAAGCCC	101.7	Martinez et al., 2018 [31]
GR Rv	CATGCTGATGAGCTACTGTTGTT		
SOD Fw	GGGCAATGCCAATAACTCCACA	104.5	Pedro et al., 2019 [30]
SOD Rv	AGGACCATGGTGATCCATGAGAAG		
GPx Fw	GAACTGCAGCAATGGTGAGA	100.3	Pedro et al., 2019 [30]
GPx Rv	CATGAGAGAGATGGGGTCGT		
HSP70-Fw	AGGGAACGCAACGTCCTGATTT	102	Vargas-Chacoff et al., 2019 [32]
HSP70-Rv	ACTAACCAGGCGGTTGTCAAAGTC		
HSP90-Fw	CATTCGTGGAACGCCTTCGAAA	101	Vargas-Chacoff et al., 2019 [32]
HSP90-Rv	AGAGACAAGGGTCTTGCCGTCATA		
18S Fw	GTCCGGGAAACCAAAGTC	103.5	Pedro et al., 2019 [30]
18S Fw	TTGAGTCAAATTAAGCCGCA		

## Data Availability

The raw data supporting the conclusions of this article will be made available by the authors on request.

## References

[1] McCormick S.D., McCormick S.D., Farrell A.P., Brauner C. (2013). Smolt physiology and endocrinology. Euryhaline Fishes.

[2] Fry F.E. (1947). Effects of the Environment on Animal Activity Biological Series.

[3] Saravia J., Nualart D., Paschke K., Pontigo J.P., Navarro J.M., Vargas-Chacoff L. (2024). Temperature and immune challenges modulate the transcription of genes of the ubiquitin and apoptosis pathways in two high-latitude Notothenioid fish across the Antarctic Polar Front. Fish Physiol. Biochem..

[4] Vargas-Chacoff L., Moneva F., Oyarzún R., Martínez D., Munoz J.L.P., Bertran C., Mancera J.M. (2014). Environmental salinity-modified osmoregulatory response in the sub-Antarctic notothenioid fish *Eleginops maclovinus*. Polar Biol..

[5] Vargas-Chacoff L., Regish A.M., Weinstock A., McCormick S.D. (2018). Effects of elevated temperature on osmoregulation and stress responses in Atlantic salmon *Salmo salar* smolts in fresh water and seawater. J. Fish Biol..

[6] Vargas-Chacoff L., Arjona F.J., Ruiz-Jarabo I., García-López A., Flik G., Mancera J.M. (2020). Water temperature affects osmoregulatory responses in gilthead sea bream (*Sparus aurata* L.). J. Therm. Biol..

[7] Vargas-Chacoff L., Regish A.M., Bjornsson B.T., McCormick S.D. (2021). Effects of long-term cortisol treatment on growth and osmoregulation of Atlantic salmon and brook trout. Gen. Comp. Endocrinol..

[8] Vargas-Chacoff L., Martínez D., Oyarzún R., Paschke K., Navarro J.M. (2021). The osmotic response capacity of the Antarctic fish *Harpagifer antarcticus* is insufficient to cope with projected temperature and salinity under climate change. J. Therm. Biol..

[9] Vargas-Chacoff L., Dann F., Oyarzún-Salazar R., Nualart D., Muñoz J.L.P. (2025). Oxidative Stress Response of Liver Cell Culture in Atlantic Salmon Challenged Under Two Antibiotics: Oxytetracycline and Florfenicol. Toxics.

[10] Nualart D., Paschke K., Guerreiro P.M., McCormick S.D., González-Wevar C., Cheng C.C.-H., Vargas Chacoff L. (2025). Combined effects of PVC microplastics and thermal rise alter the oxidative stress response in Antarctic fish Harpagifer antarcticus and Sub-Antarctic *Harpagifer bispinis*. Mar. Pollut. Bull..

[11] Rand G.M. (1995). Fundamentals of Aquatic Toxicology: Effects, Environmental Fate and Risk Assessment.

[12] Van der Oost R., Beyer J., Vermeulen N.P.E. (2003). Fish bioaccumulation and biomarkers in environmental risk assessment: A review. Environ. Toxicol. Pharmacol..

[13] Borković S.S., Šaponjić J.S., Pavlović S.Z., Blagojević D.P., Milošević S.M., Kovačević T.B., Radojičić R.M., Spasić M.B., Žikić R.V., Saičić Z.S. (2005). The activity of antioxidant defences enzymes in the mussel *Mytilus galloprovincialis* from the Adriatic Sea. Comp. Biochem. Physiol. C.

[14] Elia A.C., Anastasi V., Martin Dörr A.J. (2006). Hepatic antioxidant enzymes and total glutathione of *Cyprinus carpio* exposed to three disinfectants, chlorine dioxide, sodium hypochlorite and peracetic acid, for superficial water potabilization. Chemosphere.

[15] Winston G.W., Di Giulio R.T. (1991). Prooxidant and antioxidant mechanisms in aquatic organisms. Aquat. Toxicol..

[16] Soldatov A.A., Gostyukhina O.L., Golovina I.V. (2007). Antioxidant enzymes compplex of tissues of the bivalve Mytilus galloprovincialis Lam. Under normal and oxidative-stress conditions: A review. Appl. Biochem. Microbiol..

[17] FAO (2024). The State of World Fisheries and Aquaculture 2024. Sustainability in Action.

[18] SERNAPESCA (2023). Report with Health Background of Freshwater and Sea Year 1st Semester.

[19] Christensen A.M., Ingerslev F., Baun A. (2006). Ecotoxicity of mixtures of antibiotics used in aquacultures. Environ. Toxicol. Chem..

[20] Avendaño-Herrera R., Mancilla M., Miranda C.D. (2023). Use of antimicrobials in Chilean Salmon farming: Facts, myths and perspectives. Rev. Aquac..

[21] Elema M.O., Hoff K.A., Kristensen H.G. (1996). Bioavailability of oxytetracycline from medicated feed administered to Atlantic salmon (*Salmo salar* L.) in seawater. Aquaculture.

[22] Nualart D., Muñoz J.L.P., Vargas-Chacoff L. (2025). Intestinal Immune System Expression of Coho Salmon Challenged with Oxytetracycline: In Vivo and In Vitro Approach. Int. J. Mol. Sci..

[23] Muñoz J.L.P., Martínez D., Nualart D.P., Mardones O., Delmoral I., Morera F., Vargas-Chacoff L. (2025). Antibiotic oxytetracycline is affecting the dynamics of serotonergic response in brain of coho salmon. Aquaculture.

[24] Schnell S., Kawano A., Porte C., Lee L.E.J., Bols N.C. (2008). Effects of Ibuprofen on the Viability and Proliferation of Rainbow Trout Liver Cell Lines and Potential Problems and Interactions in Effects Assessment. Environ. Toxicol. Int. J..

[25] Pontigo J.P., Vargas-Chacoff L. (2021). Growth hormone (GH) and growth hormone release factor (GRF) modulate the immune response in the SHK-1 cell line and leukocyte cultures of head kidney in Atlantic salmon. Gen. Comp. Endocrinol..

[26] Nualart D.P., Dann F., Oyarzun-Salazar R., Morera F.J., Vargas-Chacoff L. (2023). Immune Transcriptional Response in Head Kidney Primary Cell Cultures Isolated from the Three Most Important Species in Chilean Salmonids Aquaculture. Biology.

[27] Tafalla C., Novoa B., Alvarez J.M., Figueras A. (1999). In vivo and in vitro effect of oxytetracycline treatment on the immune response of turbot, *Scophthalmus maximus* (L.). J. Fish Dis..

[28] Livak K.J., Schmittgen T.D. (2001). Analysis of relative gene expression data using realtime quantitative PCR and the 2^−ΔΔct^ method. Methods.

[29] Rasmussen R., Meuer S., Wittwer C., Nakagawara K. (2001). Quantification on the Light Cycler. Rapid Cycle Real-Time PCR, Methods and Applications.

[30] Pedro A.V.F., Martínez D., Pontigo J.P., Vargas-Lagos C., Hawes C., Wadsworth S., Morera F.J., Vargas-Chacoff L., Yáñez A.J. (2019). Transcriptional activation of genes involved in oxidative stress in *Salmo salar* challenged with *Piscirickettsia salmonis*. Comp. Biochem. Physiol. B.

[31] Martínez D., Vargas-Lagos C., Oyarzún R., Loncoman C.A., Pontigo J.P., Yáñez A.J., Vargas-Chacoff L. (2018). Temperature modulates the immunological response of the sub-antarctic notothenioid fish *Eleginops maclovinus* injected with Piscirickettsia salmonis. Fish Shellfish. Immunol..

[32] Vargas-Chacoff L., Muñoz J.L.P., Saravia J., Oyarzún R., Pontigo J.P., González M.P., Mardones O., Hawes C., Pino J., Wadsworth S. (2019). Neuroendocrine stress response in Atlantic salmon (*Salmo salar*) and Coho salmon (*Oncorynchus kisutch*) during sea lice infestation. Aquaculture.

[33] Aebi H. (1984). Catalase in vitro. Methods in Enzymology.

[34] Lopez-Galindo C., Vargas-Chacoff L., Nebot E., Casanueva J.F., Rubio D., Sole M., Mancera J.M. (2010). Sublethal effects of the organic antifoulant Mexel (R) 432 on osmoregulation and xenobiotic detoxification in the flatfish *Solea senegalensis*. Chemosphere.

[35] Carlberg I., Mannervik B. (1984). Glutathione reductase. Methods in Enzymology.

[36] Flohe L., Gunzler G.A. (1984). Assays of glutathione peroxidase. Methods in Enzymology.

[37] Sun Y., Oberley L.W., Li Y. (1988). A simple method for clinical assay of superoxide dismutase. Clin. Chem..

[38] Veith A., Moorthy B. (2018). Role of cytochrome P450s in the generation and metabolism of reactive oxygen species. Curr. Opin. Toxicol..

[39] Sies H., Berndt C., Jones D.P. (2017). Oxidative stress. Annu. Rev. Biochem..

[40] Gheorghe S., Stan M.S., Mitroi D.N., Staicu A.C., Cicirma M., Lucaciu I.E., Nita-Lazar M., Dinischiotu A. (2022). Oxidative Stress and Histopathological Changes in Gills and Kidneys of *Cyprinus carpio* following Exposure to Benzethonium Chloride, a Cationic Surfactant. Toxics.

[41] Solhaug A., Gjessing M., Sandvik M., Eriksen G.S. (2023). The gill epithelial cell lines RTgill-W1, from Rainbow trout and ASG-10, from Atlantic salmon, exert different toxicity profiles towards rotenone. Cytotechnology.

[42] Ramesh M., Sujitha M., Anila P.A., Ren Z., Poopal R.K. (2021). Responses of *Cirrhinus mrigala* to second-generation fluoroquinolone (ciprofloxacin) toxicity: Assessment of antioxidants, tissue morphology, and inorganic ions. Environ. Toxicol..

[43] Liang X., Wang L., Ou R., Nie X., Yang Y., Wang F., Li K. (2015). Effects of norfloxacin on hepatic genes expression of P450 isoforms (CYP1A and CYP3A), GST and P-glycoprotein (P-gp) in Swordtail fish (*Xiphophorus Helleri*). Ecotoxicology.

[44] Iftikhar N., Zafar R., Hashmi I. (2022). Multi-biomarkers approach to determine the toxicological impacts of sulfamethoxazole antibiotic on freshwater fish *Cyprinus carpio*. Ecotoxicol. Environ. Saf..

[45] Bainy A.C.D., Saito E., Carvalho P.S.M., Junqueira V.B.C. (1996). Oxidative stress in gill, erythrocytes, liver and kidney of Nile tilapia *(Oreochromis niloticus)* from a polluted site. Aquat. Toxicol..

[46] Antão-Barboza L.G., Russo Vieira L., Branco V., Carvalho C., Guilhermino L. (2018). Microplastics increase mercury bioconcentration in gills and bioaccumulation in the liver, and cause oxidative stress and damage in *Dicentrarchus labrax* juveniles. Sci. Rep..

[47] Regoli F., Winston G.W., Gorbi S., Frenzilli G., Nigro M., Corsi I., Focardi S. (2003). Integrating enzymatic responses to organic chemical exposure with total oxyradical absorbing capacity and DNA damage in the European eel *Anguilla anguilla*. Environ. Toxicol. Chem..

[48] Regoli F., Nigro M., Benedetti M., Gorbi S., Pretti C., Gervasi P.G., Fattorini D. (2005). Interactions between metabolism of trace metals and xenobiotic agonists of the aryl hydrocarbon receptor in the Antarctic fish *Trematomus bernacchii*: Environmental perspectives. Environ. Toxicol. Chem..

[49] Benedetti M., Martuccio G., Fattorini D., Canapa A., Barucca M., Nigro M., Regoli F. (2007). Oxidative and modulatory effects of trace metals on metabolism of polycyclic aromatic hydrocarbons in the Antarctic fish *Trematomus bernacchii*. Aquat. Toxicol..

[50] Benedetti M., Fattorini D., Martuccio G., Nigro M., Regoli F. (2009). Interactions between trace metals (Cu, Hg, Ni, Pb) and 2,3,7,8-tetrachlorodibenzo-p-dioxin in the Antarctic fish *Trematomus bernacchii*: Oxidative effects on biotransformation pathway. Environ. Toxicol. Chem..

[51] Vakharia D.D., Liu N., Pause R., Fasco M., Bessette E., Zhang Q.-Y., Kaminsky L.S. (2001). Effect of metals on polycyclic aromatic hydrocarbon induction of CYP1A1 and CYP1A2 in human hepatocyte cultures. Toxicol. Appl. Pharmacol..

[52] Caipang C.M.A., Lazado C.C., Brinchmann M.F., Berg I., Kiron V. (2009). In vivo modulation of immune response and antioxidant defense in Atlantic cod, *Gadus morhua* following oral administration of oxolinic acid and florfenicol. Comp. Biochem. Physiol. Part. C.

[53] Kohen R., Nyska A. (2002). Oxidation of biological systems: Oxidative stress phenomena, antioxidants, redox reactions, and methods for their quantification. Toxicol. Pathol..

[54] García-Pimentel M.M., Mezzelani M., Valdés N.J., Giuliani M.E., Gorbi S., Regoli F., León V.M., Campillo J.A. (2025). Integrative oxidative stress biomarkers in gills and digestive gland of the combined exposure to citalopram and bezafibrate with polyethylene microplastics on mussels *Mytilus galloprovincialis*. Environ. Pollut..

[55] Rodrigues S., Antunes S.C., Correia A.T., Nunes B. (2017). Rainbow trout (*Oncorhynchus mykiss*) pro-oxidant and genotoxic responses following acute and chronic exposure to the antibiotic oxytetracycline. Ecotoxicology.

[56] Yilmaz S., Atessahin A., Sahna E., Karahan I., Ozer S. (2006). Protective effect of lycopene on adriamycin-induced cardiotoxicity and nephrotoxicity. Toxicology.

[57] Gibson B.W. (2005). The human mitochondrial proteome: Oxidative stress protein modifications and oxidative phosphorylation. Int. J. Biochem. Cell Biol..

[58] Gnanasoundari M., Pari L. (2006). Impact of naringenin on oxytetracycline-mediated oxidative damage in kidney of rats. Ren. Fail..

[59] Álvarez-Muñoz D., Gómez-Parra A., Blasco J., Sarasquete C., González-Mazo E. (2009). Oxidative stress and histopathology damage related to the metabolism of dodecylbenzene sulfonate in Senegalese sole. Chemosphere.

[60] Feng T., Li Z.B., Guo X.Q., Guo J.P. (2008). Effects of trichlorfon and sodium dodecyl sulphate on antioxidant defense system and acetylchloniesterase of Tilapia nilotica in vitro. Pest. Biochem. Phys..

[61] Kładna A., Michalska T., Berczýnski P., Kruk I., Aboul-Enein H.Y. (2012). Evaluation of the antioxidant activity of tetracycline antibiotics in vitro. Luminescence.

[62] Vargas-Chacoff L., Astola A., Arjona F.J., Martín del Río M.P., García-Cózar F., Mancera J.M., Martínez-Rodríguez G. (2009). Pituitary gene and protein expression under experimental variation on salinity and temperature in gilthead sea bream *Sparus aurata*. Comp. Biochem. Physiol. B.

[63] Iwama G., Afonso L., Todgham A., Ackerman P., Nakano K. (2004). Are HSPs suitable for indicating stressed states in fish?. J. Exp. Biol..

[64] Fowler S., Hamilton D., Currie S. (2009). A comparison of the heat shock response in juvenile and adult rainbow trout (*Oncorhynchus mykiss*)—Implications for increased thermal sensitivity with age. Can. J. Fish. Aquat. Sci..

[65] Hori T.S., Gamperl A.K., Afonso L.O.B., Johnson S.C., Hubert S., Kimball J., Bowman S., Rise M.L. (2010). Heat-shock responsive genes identified and validated in Atlantic cod (*Gadus morhua*) liver, head kidney and skeletal muscle using genomic techniques. BMC Genom..

[66] LeBlanc S., Höglund E., Gilmour K.M., Currie S. (2012). Hormonal modulation of the heat shock response: Insights from fish. Am. J. Physiol. Regul. Integr. Comp. Physiol..

[67] LeBlanc S., Middleton S., Gilmour K.M., Currie S. (2011). Chronic social stress impairs thermal tolerance in the rainbow trout (*Oncorhynchus mykiss*). J. Exp Biol..

[68] Niforou K., Cheimonidou C., Trougakos I.P. (2014). Molecular chaperones and proteostasis regulation during redox imbalance. Redox Biol..

[69] Martínez D., Vargas-Lagos C., Saravia J., Oyarzún R., Loncoman C., Pontigo J.P., Vargas-Chacoff L. (2020). Cellular stress responses of *Eleginops maclovinus* fish injected with *Piscirickettsia salmonis* and submitted to thermal stress. Cell Stress Chaperones.

